# Choice of Reference Sequence and Assembler for Alignment of *Listeria monocytogenes* Short-Read Sequence Data Greatly Influences Rates of Error in SNP Analyses

**DOI:** 10.1371/journal.pone.0104579

**Published:** 2014-08-21

**Authors:** Arthur W. Pightling, Nicholas Petronella, Franco Pagotto

**Affiliations:** 1 Listeriosis Reference Service for Canada, Research Division, Bureau of Microbial Hazards, Food Directorate, Health Products and Food Branch, Health Canada, Ottawa, Ontario, Canada; 2 Biostatistics and Modelling Division, Bureau of Food Surveillance and Science Integration, Food Directorate, Health Products and Food Branch, Health Canada, Ottawa, Ontario, Canada; University of Westminster, United Kingdom

## Abstract

The wide availability of whole-genome sequencing (WGS) and an abundance of open-source software have made detection of single-nucleotide polymorphisms (SNPs) in bacterial genomes an increasingly accessible and effective tool for comparative analyses. Thus, ensuring that real nucleotide differences between genomes (*i.e.*, true SNPs) are detected at high rates and that the influences of errors (such as false positive SNPs, ambiguously called sites, and gaps) are mitigated is of utmost importance. The choices researchers make regarding the generation and analysis of WGS data can greatly influence the accuracy of short-read sequence alignments and, therefore, the efficacy of such experiments. We studied the effects of some of these choices, including: i) depth of sequencing coverage, ii) choice of reference-guided short-read sequence assembler, iii) choice of reference genome, and iv) whether to perform read-quality filtering and trimming, on our ability to detect true SNPs and on the frequencies of errors. We performed benchmarking experiments, during which we assembled simulated and real *Listeria monocytogenes* strain 08-5578 short-read sequence datasets of varying quality with four commonly used assemblers (BWA, MOSAIK, Novoalign, and SMALT), using reference genomes of varying genetic distances, and with or without read pre-processing (*i.e.*, quality filtering and trimming). We found that assemblies of at least 50-fold coverage provided the most accurate results. In addition, MOSAIK yielded the fewest errors when reads were aligned to a nearly identical reference genome, while using SMALT to align reads against a reference sequence that is ∼0.82% distant from 08-5578 at the nucleotide level resulted in the detection of the greatest numbers of true SNPs and the fewest errors. Finally, we show that whether read pre-processing improves SNP detection depends upon the choice of reference sequence and assembler. In total, this study demonstrates that researchers should test a variety of conditions to achieve optimal results.

## Introduction

Comprehensive sequencing and analysis of bacterial genomes are increasingly valuable tools in fields such as epidemiology [1]–[3], population genetics [4], [5], and experimental evolution [6]. Advances in DNA sequencing technologies and reduced costs have made high-quality whole-genome sequence (WGS) data readily available [7], while an abundance of open-source software has made it possible for whole-genome analyses to be performed in individual laboratories [8]. In particular, single-nucleotide polymorphism (SNP) discovery is useful for distinguishing bacterial lineages [9] and SNPs may serve as markers for phenotypic characteristics such as antibiotic resistance [10]. Putative SNPs are most commonly identified by using a fully sequenced (closed) reference genome as a guide to assemble large numbers of short sequence reads (reference-guided assembly) and searching for nucleotide differences between reference and draft genome sequences [11]. SNP analyses can be performed with *de-novo* assemblies. However, increased computational requirements and slow processing times relative to reference-guided assemblies may make them impractical [12]. In addition, assemblies performed against references often yield more data than *de-novo* assemblies, especially when sequence coverage is low [13]. Although inaccuracies in reference-guided short-read sequence alignments may arise due to inherent errors associated with a given sequencing technology or the quality of DNA extractions and library preparations, such events are more likely to arise from misassembled reads [14], especially if appropriate pre- and post-processing of reads have been performed such as read-quality trimming and filtering and local realignments around indels [11], [15], [16]. Furthermore, the genetic distances between reference and subject sequences are likely to effect SNP detection as more distant references may provide additional challenges for reference-guided assemblers [12].

Far from there being a standard method for assembling WGS data, there is currently a wealth of software available that make different assumptions that are likely to influence the final assemblies and, therefore, the accurate identification of SNPs. Reference-guided sequence assembly software builds alignments of short sequence reads, assessing the placement of each read by calculating the probability of its match with the reference, while SNP identification (SNP calling) is performed by programs that use a combination of coverage statistics and estimated error rates of the sequencing platforms used [17], [18]. Reads that have been misaligned by the assembler may confound the SNP calling software, manifesting as misidentified SNPs (*i.e.*, false positive calls), an inability of the software to make calls at all (*i.e.*, ambiguous sites), or the inappropriate introduction of gaps into consensus sequences. In addition to causing problems with correlation analyses or bacterial typing assays that are based upon WGS data (such as *in silico* multi-locus sequence typing), errors may result in distortion of estimates of the genetic distances between sequences and may influence phylogenetic analyses. Therefore, decisions such as the selection of a reference-guided assembler, selection of an appropriate reference sequence, and depth of sequence coverage should be carefully considered when designing any experiment involving SNP identification or the use of consensus sequences for downstream analyses.

Using the well studied pathogenic bacterium *Listeria monocytogenes* [19] as an example, we present a set of benchmarking experiments performed on simulated Illumina short-read sequence data and, because simulated reads may not always accurately represent errors that appear in actual datasets, we assembled reads obtained from sequencing runs of varying qualities performed on an Illumina MiSeq benchtop sequencer. We measured the numbers of true SNP differences, false positive SNPs, ambiguous sites, and gaps introduced into draft chromosome sequences under a variety of conditions, including: i) a range of sequencing coverage; ii) the use of four reference-guided sequence assemblers (Burrows-Wheeler Aligner [20], Novoalign, MOSAIK, and SMALT), selected due to their popularity, accessibility and use of different algorithms (Burrows-Wheeler transform [21], global Needleman-Wunsch [22], banded Smith-Waterman and a combination of short-word hashing and Smith-Waterman [23], [24], respectively); iii) the use of reference sequences of different genetic distances, and iv) quality filtering and trimming of reads prior to assembly. Analyses were performed with two highly clonal strains of *L. monocytogenes* (08-5578 [2], [3] and EGD-e [25]). The chromosome sequences of *L. monocytogenes* strains 08-5578 and EGD-e are approximately 3.11 and 2.94 Mb in length, respectively. Both chromosomes have an average GC content of 38% and experience few chromosomal rearrangements [26], [27].

## Materials and Methods

### DNA extraction, library construction, and DNA sequencing

A *Listeria monocytogenes* strain 08-5578 isolate frozen in glycerol was streaked on pre-warmed Tryptose Agar plates and incubated at 37°C over-night. A single colony was picked and used to inoculate 5 ml pre-warmed Brain Heart Infusion (BHI) broth and incubated over-night at 37°C with shaking (200 rpm). Then, 200 µl of the culture was transferred to 50 ml pre-warmed BHI and incubated at 37°C with shaking for 6 hours to achieve the mid-logarithmic growth phase [29], [30]. Approximately 25 ml of culture was decanted into a 50 ml falcon tube and centrifuged at 3800 RCF for 5 minutes. The pellet was completely dissolved in 500 µl Tris-ethylenediaminetetraacetic acid by vortexing. We added 500 µl phenol-chloroform (1∶1), 30 µl sodium acetate (3M, pH 5.2), and 30 µl sodium dodecyl sulfate and mixed vigorously by shaking. The entire mixture was then pipetted into a 2 ml screw-cap tube filled with approximately 0.5 ml glass beads (0.1 mm). The tube was shaken in a Mini-Beadbeater machine (BioSpec products, Bartlesville, Oklahoma) for 45 seconds using the “Homogenizer” setting and placed on ice for 45 seconds. Shaking was repeated an additional four times. Approximately 300 µl of the mixture was then added to a Maxwell 16 Cell DNA Purification Kit cartridge and the sample was run using the standard DNA Blood/Cells protocol on a Maxwell 16 machine (Promega, Madison, Wisconsin) with elution in 300 µl nuclease-free water. RNA contamination was removed by adding 2 µl RNase A (Qiagen Sciences, Maryland) and incubating the sample for 10 minutes at 37°C. A single phenol-chloroform-isoamyl alcohol (25∶24∶1) extraction followed by two ethanol precipitations was done. The sample was split into four subsamples. Each subsample was indexed with Nextera XT DNA Sample Preparation Kits (Illumina, San Diego, California) according to the standard protocol and sequenced (2×250 bp reads) on a MiSeq benchtop sequencer (Illumina) three separate times for a total of twelve sets of short-read sequences. These data have been deposited to the National Center for Biotechnology Information (NCBI) Sequence Read Archive (SRA) under accession numbers SRR1342176, SRR1342220, SRR1373524, SRR1373525, SRR1373527, SRR1373529, SRR1373530, SRR1373531, SRR1373534, SRR1373535, SRR1507228, and SRR1508282.

### Assembly of short-read sequence data

An automated bioinformatic pipeline was written using the Perl programming language to execute applications for the quality assessment, pre-processing, assembly, and analysis of all sequencing reads. In order to ensure that only the highest quality data was used for assembly, reads were trimmed and filtered with PoPoolation [31] set to a minimum length of 50 bp and a quality score threshold of 20. Global mapping of reads was then performed with each of four reference-guided short-read sequence assemblers: Burrows-Wheeler aligner v0.6.1-r104 [20], MOSAIK v2.1 (code.google.com/p/mosaik-aligner/), Novoalign v3.00.03 (novocraft.com/main/index.php), and SMALT v0.7.4 (sanger.ac.uk/resources/software/smalt/). We used the Genome Analysis Toolkit [32] to perform local realignments around indels according to GATK Best Practices recommendations [16]. The calls used for each assembler and the GATK software are included in Table S1. We then used the SAMtools/BCFtools package [33] to identify SNPs and calculate consensus sequences. All analyses were performed with an AMD Phenom II X6 1090T processor and 16 GB of DDR3 RAM.

### Construction of simulated reads

Ten sets of simulated 150 bp Illumina paired-end reads were generated with ART v1.5.0 [28] to 50-fold coverage using a *Listeria monocytogenes* strain 08-5578 chromosome sequence obtained from NCBI (NC_013766.1) as a reference. Simulated nucleotide substitutions (10^1^–10^5^) were introduced *in silico* at random positions in the 08-5578 chromosome sequence to generate five “mutated” reference genomes with a Perl script (SNP_insert.pl) available at http://sourceforge.net/projects/snpinsert/files/. The ten sets of reads were then assembled as described above to each of the five modified reference sequences with BWA, MOSAIK, Novoalign, and SMALT.

### Phylogenetic analysis and measurement of genetic distances

We assembled short-read sequence data from the best of twelve runs of *Listeria monocytogenes* strain 08-5578 genomic DNA on an Illumina MiSeq benchtop sequencer with BWA, MOSAIK, Novoalign, and SMALT using both NCBI strains 08-5578 and EGD-e chromosome sequences as references and we calculated consensus sequences as described above. We then aligned the consensus sequences with progressiveMauve v2.3.1 [34]. The alignment was curated with Gblocks v0.91b [35], [36] using the default settings. Phylogenetic trees were calculated with the Randomized Axelerated Maximum Likelihood tool [37]; using the GTRGAMMAI model and 25 gamma categories to generate and select the most likely of 100 bootstrap replicates. Pairwise nucleotide distances were calculated with the same alignment using PHYLIP v3.6 to apply the Jukes-Cantor method with gamma-distributed weights across sites.

## Results and Discussion

We assessed the efficacy of four commonly used reference-guided short-read sequence assemblers (BWA, MOSAIK, Novoalign, and SMALT) to generate alignments suitable for accurate detection of single-nucleotide polymorphisms (SNPs) using both simulated reads and actual reads obtained from sequencing runs of *Listeria monocytogenes* strain 08-5578 genomic DNA on an Illumina MiSeq benchtop machine. Performance was measured by comparing consensus sequences calculated from each of the resulting assemblies with completely sequenced references and counting the numbers of known nucleotide differences between the subjects and references (true positive SNPs), incorrectly called nucleotides (false positive SNPs), ambiguously called sites, and gaps. Here, ambiguously called sites were considered to be artefacts of genome sequencing and assembly, rather than indicators of heterogeneity, as *L. monocytogenes* genomes are highly conserved [38] with low evolutionary rates [39]. In total, ten sets of simulated reads were generated at 50-fold coverage using the 08-5578 chromosomal DNA sequence (NC_013766.1) available from the National Center for Biotechnology Information (NCBI) archive as a template. In order to mimic the use of reference sequences of varying genetic distances, the NCBI 08-5578 chromosome sequence was altered by randomly introducing 10^1^–10^5^ variants *in silico*, generating five reference sequences that are approximately 0.00032–3.2% distant at the nucleotide level from the unaltered 08-5578 chromosome sequence. All ten sets of simulated reads were then aligned with each assembler using each of the five altered chromosome sequences as references.

**Figure 1:**
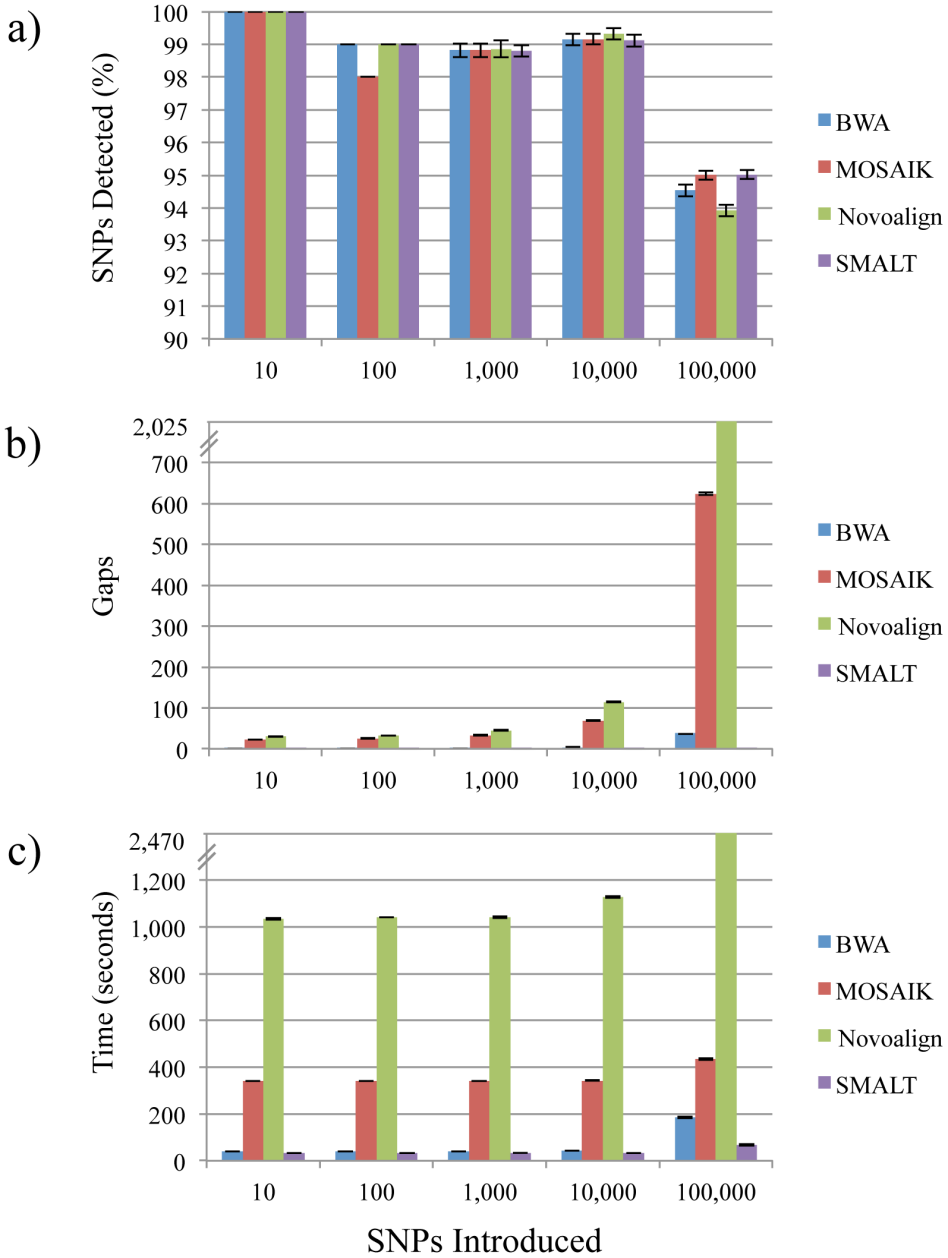
Comparison of consensus sequences calculated from assemblies of simulated Illumina short-read data aligned to references of different genetic distances with four reference-guided assemblers. Ten sets of simulated sequencing reads were generated using a *Listeria monocytogenes* strain 08-5578 chromosome sequence obtained from the National Center for Biotechnology Information archive as a reference. Nucleotide variants were randomly introduced (10^1^–10^5^) *in silico* to the 08-5578 chromosome sequence to simulate the presence of SNPs in five reference sequences. The performance of four reference-guided short-read sequence assemblers (BWA, MOSAIK, Novoalign, and SMALT) was assessed by averaging the percentages of true SNPs detected (a) and the numbers of gaps present (b) in the consensus sequences generated from alignments of the ten sets of reads. In addition, average assembly processing times are provided (c).

Comparison of the resulting consensus sequences with the reference sequences revealed that, when only ten nucleotide variants were present, all SNPs were detected in every sequence regardless of which assembler was used (Figure 1a and Table S2). All four assemblers also produced comparable results (approximately 98–99% detection) when 10^2^, 10^3^, or 10^4^ variants were introduced. However, with the introduction of 10^5^ variants, simulating the use of a reference that is 3.2% distant, the frequencies of SNPs detected dropped to approximately 94–95%, illustrating the inverse relationship between SNP detection and the genetic distances of subject to reference chromosome sequences. In addition, while gaps were introduced into almost every consensus sequence, BWA and SMALT generated assemblies that resulted in fewer such events than either MOSAIK or Novoalign (Figure 1b and Table S2). Also, although the numbers of gaps increased considerably when BWA, MOSAIK, or Novoalign were used to assemble the data against the 3.2% distant reference (averaging 39.20, 624.00, and 2,024.60 gaps, respectively), the increases observed when SMALT was used were far less severe (averaging 1.90 gaps). Similarly, BWA, MOSAIK, and Novoalign required more processing time than SMALT and the amount of time necessary to assemble the short-read sequencing data increased precipitously with the presence of 10^5^ variants (Figure 1c and Table S2).

These results indicate that, with the introduction of only 10 nucleotide variants, all of the reference-guided assemblers here were equally capable of assembling the short-read sequence data, producing alignments that resulted in the detection of all SNPs and the introduction of very few (if any) gaps within consensus sequences. With the introduction of 100,000 variants, we observed significant declines in the percentages of true SNPs detected for all assemblers, increases in the numbers of gaps (especially when using MOSAIK or Novoalign), and increased processing times for all assemblers but most notably for MOSAIK and Novoalign. Interestingly, alignments of the simulated data yielded consensus sequences with only between 0 and 3 false positive SNPs and no ambiguous sites regardless of which assembler was used (data not shown). We thus hypothesized that the absence of these types of errors was most likely due to the random distribution of the *in silico* SNPs and that increases in the numbers of false positive SNPs and ambiguous sites would be observed when short-read sequence data from real sequencing runs was assembled.

In order to assess the numbers of errors introduced into consensus sequences when real short-read sequence data was assembled, we extracted the genomic DNA of the Listeriosis Reference Service for Canada's (LRS) subculture of *L. monocytogenes* strain 08-5578. We then split the DNA sample into four subsamples and performed three sequencing runs of varying quality on a MiSeq benchtop sequencer (as indicated by cluster densities, total output, numbers of reads generated, and the numbers of reads passing filter), yielding a total of twelve sets of short-read sequence data (Table S3). We then used the chromosome sequences of both NCBI strains 08-5578 and EGD-e (NC_003210.1) as references. During the course of this experiment, we discovered that the NCBI strain 08-5578 chromosome sequence submission is different from the LRS strain 08-5578 chromosome sequence at three nucleotide positions (1,329,720; 2,870,261; and 2,870,308), making them ∼0.000096% distant. These differences were verified with Sanger sequencing (data not shown). The strain EGD-e chromosome sequence has 25,347 nucleotide differences compared to the LRS 08-5578 chromosome sequence (∼0.82% distant). So, we were able to test the abilities of the short-read reference-guided sequence assemblers to generate alignments that resulted in consensus sequences that included all true positive sites and we were able to assess the rates of error by counting any false positive sites, ambiguous sites, or gaps.

**Figure 2:**
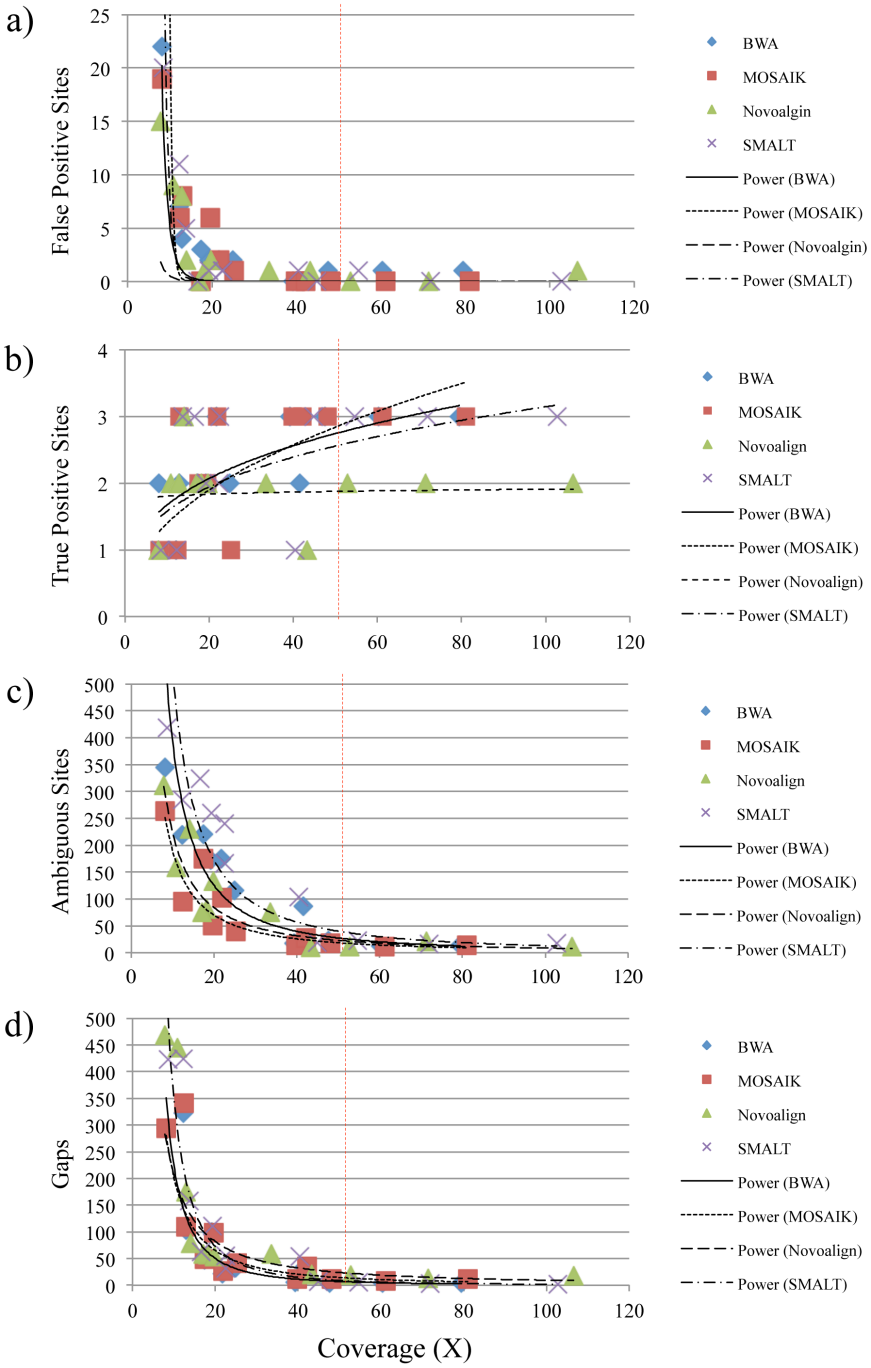
Comparison of consensus sequences calculated from alignments of Illumina MiSeq short-read data to a nearly identical reference with four reference-guided assemblers. Genomic DNA from the Listeriosis Reference Service for Canada's (LRS) *Listeria monocytogenes* strain 08-5578 subculture was indexed and sequenced twelve times. The resulting reads were aligned with four reference-guided assemblers (BWA, MOSAIK, Novoalign, and SMALT) using an *L. monocytogenes* strain 08-5578 chromosome sequence obtained from the National Center for Biotechnology Information (NCBI) archive as a reference. The NCBI strain 08-5578 chromosome sequence differs from the LRS strain 08-5578 chromosome sequence at three nucleotide positions. The numbers of false positive sites (a), true positive sites (b), ambiguous sites (c), and gaps (d) present in the resulting consensus sequences relative to the calculated coverage of each assembly are shown. Lines were calculated with power regression and smoothed using a Catmull-Rom Spline.

We detected between 0 and 22 false positive sites in consensus sequences calculated from alignments using the NCBI strain 08-5578 chromosome sequence as a reference and their presence seems to be correlated with decreasing coverage (Figure 2a and Table S4). The average numbers of false positive SNPs resulting from use of the different reference-guided sequence assemblers varied from 3.33 (Novoalign) to 3.75 (BWA), representing approximately 0.00011 to 0.00012% error in the consensus sequences. Among runs with at least 50-fold coverage, the numbers of false positive sites range from 0 (MOSAIK, Novoalign, and SMALT) to 1 (BWA) and represent a maximum of 0.000032% error. The numbers of true SNPs detected range from 1 to 3 for all assemblers with averages of 1.92 (Novoalign) to 2.33 (BWA, MOSAIK, and SMALT), representing 64 to 78% accuracy (Figure 2b and Table S4). Of runs with at least 50-fold coverage, Novoalign generated alignments that resulted in the detection of 2 of the 3 SNPs, while using BWA, MOSAIK, and SMALT resulted in the detection of all 3 SNPs. The majority of incorrectly called bases manifested as ambiguous sites (Figure 2c and Table S4). The average numbers of ambiguously called bases range from 90.75 to 192.25 (0.0029–0.0062% error) and from 13.00 to 19.33 (0.00042–0.00062% error) for runs of at least 50-fold coverage (MOSAIK and SMALT, respectively, in both cases). The average numbers of gaps introduced into consensus sequences range from 81.08 (BWA) to 122.25 (Novoalign) for all runs, indicating between 0.0026 and 0.0039% error in the consensus sequences, and 3.67 (SMALT) to 16.67 (Novoalign) for runs of at least 50-fold coverage, indicating between 0.00012 and 0.00054% error (Figure 2d and Table S4). Hence, errors present in consensus sequences appear to be due predominantly to low sequence coverage. However, among runs with at least 50-fold coverage, choice of assembler does influence the numbers of errors present in consensus sequences when the subject and reference are nearly identical and, in this context, assemblies calculated with MOSAIK yielded consensus sequences with the fewest errors (0.00055% total) and all 3 true nucleotide differences.

**Figure 3:**
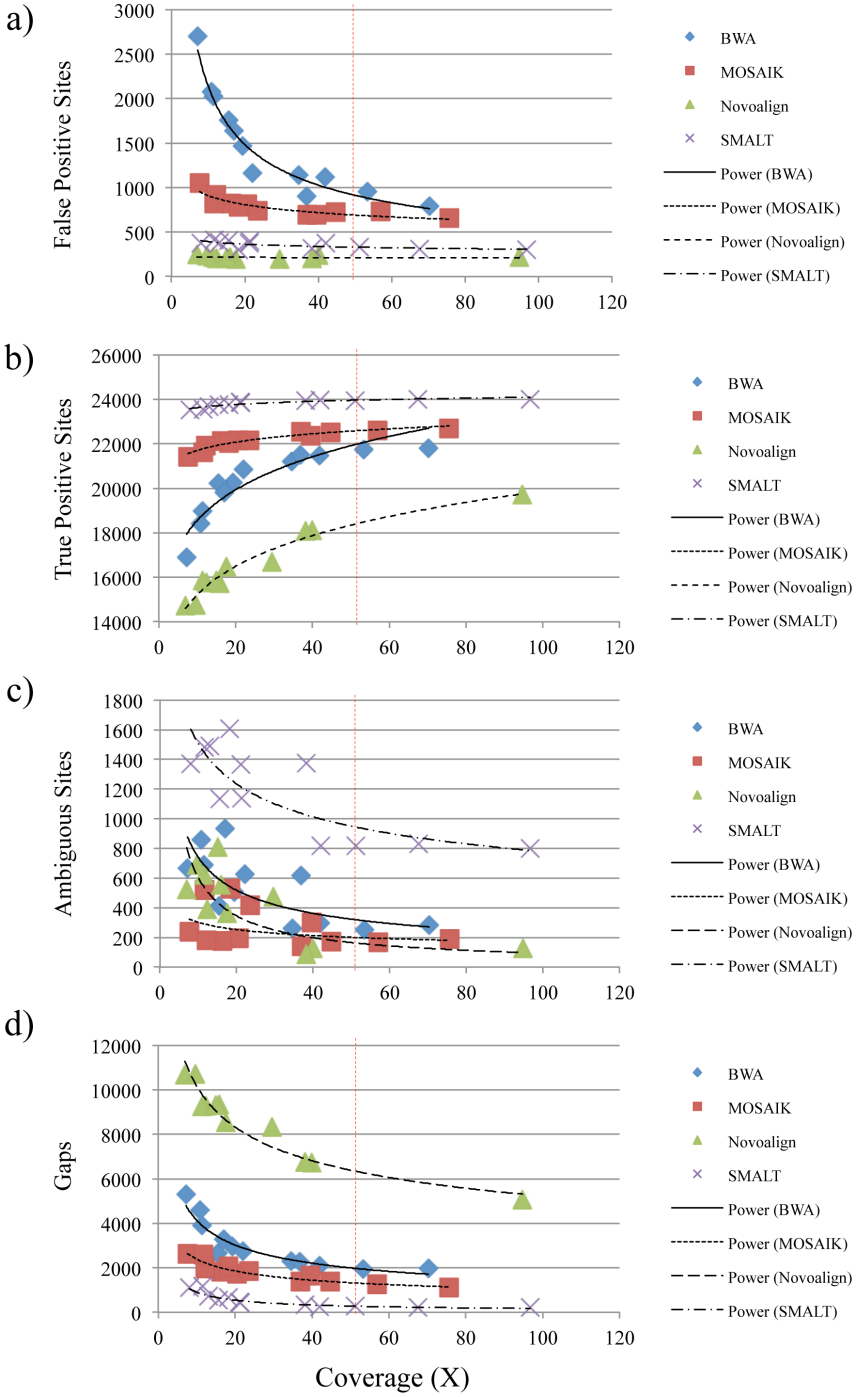
Comparison of consensus sequences calculated from alignments of Illumina MiSeq short-read data to a non-identical reference with four reference-guided assemblers. Genomic DNA from the Listeriosis Reference Service for Canada's (LRS) *Listeria monocytogenes* strain 08-5578 subculture was sequenced and indexed twelve times. The resulting reads were aligned with four reference-guided assemblers (BWA, MOSAIK, Novoalign, and SMALT) using an *L. monocytogenes* strain EGD-e chromosome sequence obtained from the National Center for Biotechnology Information (NCBI) archive as a reference. The NCBI strain EGD-e chromosome sequence differs from the LRS strain 08-5578 chromosome sequence at 25,347 nucleotide positions. The numbers of false positive sites (a), true positive sites (b), ambiguous sites (c), and gaps (d) present in the resulting consensus sequences relative to the calculated coverage of each assembly are shown. Lines were calculated with power regression and smoothed using a Catmull-Rom Spline.

To further assess the effect of reference genome selection on frequencies of errors in consensus sequences calculated from reference-guided assemblies, we aligned all twelve sets of real short-read sequence data again using the NCBI strain EGD-e chromosome sequence as a reference. The numbers of false positive sites present in these consensus sequences were substantially greater than consensus sequences calculated from assemblies performed with the nearly identical reference. Assemblies generated with Novoalign resulted in an average of 218.83 false positive sites and assemblies generated with BWA yielded an average of 1,477.17 such sites, indicating between 0.0070 and 0.047% error (Figure 3a and Table S5). Among alignments of at least 50-fold coverage, averages of 220.00 (Novoalign) to 871.50 (BWA) false positive sites were detected, representing approximately 0.0071 to 0.028% error. The numbers of true SNPs detected varied widely with the use of different assemblers, Novoalign yielded alignments that resulted in the detection of 65% of SNPs and SMALT yielded alignments that allowed for the detection of 94% of SNPs (Figure 3b and Table S5). When runs of at least 50-fold coverage are considered, between 78 and 95% of true SNPs were detected (Novoalign and SMALT, respectively). Ambiguously called sites also appeared far more frequently within these consensus sequences, averaging from 268.00 with MOSAIK to 1,186.75 with SMALT, representing between 0.0086 and 0.038% error (Figure 3c and Table S5). Among runs of at least 50-fold coverage the numbers of ambiguous sites averaged from 129.00 (Novoalign) to 817.33 (SMALT), indicating between 0.0041 and 0.026% error. The most common form of error, however, was the introduction of gaps into consensus sequences. Assemblies calculated with SMALT resulted in an average of 534.25 gaps and use of Novoalign resulted in an average of 8,549.20 gaps (0.017–0.27% error), while alignments of at least 50-fold coverage yielded averages of 245.33 (SMALT) and 5,058.00 (Novoalign), representing between 0.0079 and 0.16% error (Figure 3d and Table S5). In total, these data indicate that when using a reference that is ∼0.82% distant at the nucleotide level, although sequence coverage was important, choice of assembler contributed most significantly to the detection of true SNPs and the presence of errors. In addition, SMALT assemblies of at least 50-fold coverage generated the best consensus sequences, including an average of 95% of true SNPs and overall error rates of approximately 0.088%.

In order to determine the importance of pre-assembly processing of short-read sequence data, we counted the total numbers of SNPs and errors detected in consensus sequences calculated from assemblies of reads before and after read-quality trimming and filtering. For the real datasets aligned with the NCBI strain 08-5578 chromosome sequence, pre-processing of reads resulted in assemblies that yielded consensus sequences with fewer false positive sites than consensus sequences calculated from assemblies of unprocessed reads when BWA, MOSAIK, or SMALT were used (Figure S1a and Table S6). Alignment of pre-processed reads with Novoalign resulted in the detection of one additional false positive site. Interestingly, pre-processing of reads also resulted in a reduction in the numbers of true positive sites detected when BWA, MOSAIK, and SMALT were used, while Novoalign alignments yielded consensus sequences with the same numbers of true positive sites with and without pre-processing (Figure S1b and Table S6). The total numbers of ambiguous sites and gaps were reduced with pre-processing of reads no matter which assembler was used (Figure S1c and d and Table S6). When using the strain EGD-e chromosome sequence as a reference, quality trimming and filtering of reads increased the numbers of true SNPs detected and decreased the numbers of false positive sites, ambiguous sites, and gaps introduced into consensus sequences when BWA, MOSAIK, or SMALT were used, while quality pre-processing of reads had the opposite effect in every category when Novoalign was used (Figure S2a–d and Table S7). In total, these data indicate that whether pre-processing of reads is beneficial depends upon both the choice of reference sequence and assembler. These results are consistent with findings that read filtering and trimming do not improve the quality of assemblies or the accuracy of SNP calls [15], while others report benefits from read pre-processing [12].

Finally, in order to assess the importance of reference sequence and assembler selection to downstream applications we aligned the best of the twelve sequencing runs (according to the numbers of reads passing filter) with all four reference-guided assemblers and using chromosome sequences of both NCBI strains 08-5578 and EGD-e as references. We also phylogenetically analysed these consensus sequences (Figure S3) and measured their genetic distances (Table S8). Phylogenetic analysis reveals that there are no branches distinguishing the consensus sequences calculated from the alignments using the NCBI strain 08-5578 chromosome as a reference. Furthermore, the calculated distances between these consensus sequences are all 0.00. However, branches of various lengths separate the consensus sequences generated when the strain EGD-e chromosome sequence was used as a reference. This, of course, resulted in the grouping of sequences in the phylogenetic tree by reference and not by assembler or a random distribution of sequences. In addition, we calculated distances of 0.000078 to 0.000353 between these consensus sequences, indicating that 229 to 1,037 nucleotide differences exist. The variations in branch-lengths in the phylogenetic analysis and the measurements of genetic distances observed between these sequences incorrectly report that some sequences are more closely related than others. For example, the phylogenetic analysis indicates that the sequences in the EGD-e clade calculated from MOSAIK and Novoalign alignments share a more recent common ancestor than either shares with the sequences calculated from BWA or SMALT alignments. This result runs counter to the correct relationships illustrated by the 08-5578 clade, which show that all four sequences are equally related.

## Conclusions

Increased accessibility of whole-genome sequence data, an abundance of open-source short-read sequence assembly software, and the proven utility of SNP detection in a number of fields requires that factors that can influence the quality of assemblies and, therefore, confidence in SNP calling be carefully considered. Different types of errors, such as failures to identify true SNPs, false positive SNPs, ambiguous sites, and the introduction of gaps into consensus sequences, may arise from the sequencing process itself or may be due to the choices made on how short-read sequence data are generated and assembled. Such choices include the amount of coverage necessary to get an accurate assembly, which reference-guided short-read sequence assembler to use, which sequence to use as a reference, and whether to perform quality filtering and trimming of reads before alignment, to name just a few.

Here, we used four reference-guided sequence assemblers (BWA, MOSAIK, Novoalign, and SMALT) to align both simulated and real *Listeria monocytogenes* strain 08-5578 short-read sequence datasets. In addition, we used reference sequences that are between 0.00032 and 3.2% distant from the subjects in the case of the simulated reads and, in the case of real sequence data, we used both a reference that is different by only 3 nucleotides (strain 08-5578; ∼0.000096% distant) and a reference that differs at 25,347 nucleotide positions (strain EGD-e; ∼0.82% distant). We found that low coverage is one of the most important sources of error and that assemblies with coverage of at least 50-fold provided the best results. We found, also, that both choice of assembly software and the genetic distances of the subject sequences to the reference sequences had significant effects on SNP detection and the presence of errors in consensus sequences. Assemblers that utilize the Smith-Waterman pairwise alignment algorithm at some point during their calculations (*i.e.*, MOSAIK and SMALT) generated assemblies that yielded consensus sequences with the fewest errors and greatest numbers of true SNPs; MOSAIK performed best when the subject and reference sequences were nearly identical and SMALT performed best when the subject and reference sequences were more distant. We showed that whether pre-processing of reads influences SNP detection or the numbers of errors discovered depends upon both the assembler and reference sequence chosen. Finally, we showed that choice of reference sequence and assembler can influence downstream analyses such as measuring genetic distances and calculating phylogenetic trees.

This study demonstrates that, when planning an experiment involving reference-guided sequence assemblies, one must determine whether an appropriate reference exists. It may be a requirement for many projects that reference sequences necessary for proper reference-guided sequence assembly be closed before accurate SNP detection can occur. Also, it may be important to assess the abilities of different assemblers to align datasets and to determine whether quality filtering and trimming of reads improves the quality of draft genomes.

## Supporting Information

Figure S1 **Comparison of consensus sequences calculated from alignments of Illumina MiSeq reads to a nearly identical reference with four reference-guided sequence assemblers both before and after read-quality filtering and trimming.** *Listeria monocytogenes* strain 08-5578 genomic DNA was sequenced twelve times with an Illumina MiSeq benchtop sequencer and the resulting reads were assembled before and after read-quality filtering and trimming with four reference-guided assemblers (BWA, MOSAIK, Novoalign, and SMALT). An *L. monocytogenes* strain 08-5578 chromosome sequence obtained from the National Center for Biotechnology Information archive that differs at three nucleotide positions was used as a reference. The total numbers of false positive sites (a), true positive sites (b), ambiguous sites (c), and gaps (d) present in all consensus sequences were counted. Error bars were calculated as the square root of the standard deviation of each dataset.
(PDF)

Figure S2 **Comparison of consensus sequences calculated from alignments of Illumina MiSeq reads to a non-identical reference with four reference-guided sequence assemblers both before and after read-quality filtering and trimming.** *Listeria monocytogenes* strain 08-5578 genomic DNA was sequenced twelve times with an Illumina MiSeq benchtop sequencer and the resulting reads were assembled before and after quality filtering and trimming with four reference-guided assemblers (BWA, MOSAIK, Novoalign, and SMALT). An *L. monocytogenes* strain EGD-e chromosome sequence obtained from the National Center for Biotechnology Information archive that differs at 25,347 nucleotide positions was used as a reference. The total numbers of false positive sites (a), true positive sites (b), ambiguous sites (c), and gaps (d) present in all consensus sequences were counted. Error bars were calculated as the square root of the standard deviation of each dataset.
(PDF)

Figure S3 **Phylogenetic analysis of eight** ***Listeria monocytogenes*** **strain 08-5578 consensus sequences calculated from the alignments of four reference-guided assemblers using** ***L. monocytogenes*** **strains 08-5578 and EGD-e as references.** The best of twelve Illumina MiSeq sequencing runs of *L. monocytogenes* strain 08-5578 genomic DNA was assembled with BWA, MOSAIK, Novoalign, and SMALT using chromosome sequences of both *L. monocytogenes* strains 08-5578 and EGD-e (∼0.000096% and ∼0.82% distant from the subject at the nucleotide level, respectively), available from the National Center for Biotechnology Information archive, as references. Trees were calculated from 2,735,325 aligned nucleotides with the Randomized Axelerated Maximum Likelihood tool (RAxML; GTRGAMMA+25+I). The best of 100 bootstrap replicates is shown.
(PDF)

Table S1 **Calls used for read-quality trimming and filtering, assembly, and local realignment of Illumina short-read sequences.**
(PDF)

Table S2 **Numbers of SNPs detected and gaps present in consensus sequences calculated from assemblies of simulated short-read sequence data aligned to references of different genetic distances with four reference-guided assemblers.** Ten sets of simulated sequencing reads were generated using a *Listeria monocytogenes* strain 08-5578 chromosome sequence obtained from the National Center for Biotechnology Information archive as a reference. Nucleotide variants were randomly introduced (10^1^–10^5^) *in silico* to the 08-5578 chromosome sequence to simulate the presence of SNPs in five reference sequences. The performance of four reference-guided short-read sequence assemblers (BWA, MOSAIK, Novoalign, and SMALT) was assessed by counting the numbers of true SNPs detected and the numbers of gaps present in the consensus sequences generated from alignments of the ten sets of reads. In addition, assembly processing times are provided. The ranges of sites observed are shown with averages in parenthesis. The best values for each category are bolded.
(PDF)

Table S3 **Summary statistics describing three sequencing runs of four** ***Listeria monocytogenes*** **strain 08-5578 genomic DNA samples on an Illumina MiSeq benchtop sequencer.** Genomic DNA was extracted from an *L. monocytogenes* strain 08-5578 culture grown from a single colony. The sample was then divided into four subsamples that were indexed and sequenced three times.
(PDF)

Table S4 **Numbers of false positive sites, true positive sites, ambiguous sites, and gaps detected in consensus sequences calculated from alignments of Illumina short-read data to a nearly identical reference with four reference-guided assemblers.** The ability of four reference-guided short-read sequence assemblers (BWA, MOSAIK, Novoalign, and SMALT) to align *Listeria monocytogenes* strain 08-5578 genome sequence data was assessed by aligning twelve sets of reads to a reference chromosome sequence that differs by three nucleotides. The ranges of events observed are shown with averages in parentheses. The values for all twelve datasets are provided as well as those with 50-fold or greater coverage. The best values for each category are bolded.
(PDF)

Table S5 **Numbers of false positive sites, true positive sites, ambiguous sites, and gaps detected in consensus sequences calculated from alignments of Illumina short-read data to a non-identical reference with four reference-guided assemblers.** The ability of four reference-guided short-read sequence assemblers (BWA, MOSAIK, Novoalign, and SMALT) to align *Listeria monocytogenes* strain 08-5578 genome sequence data was assessed by aligning twelve sets of reads to a reference chromosome sequence (strain EGD-e) that differs by 25,347 nucleotides. The ranges of events observed are shown with averages in parentheses. The values for all twelve datasets are provided as well as those with 50-fold or greater coverage. The best values for each category are bolded.
(PDF)

Table S6 **Total numbers of false positive sites, true positive sites, ambiguous sites, and gaps detected in consensus sequences calculated from alignments of Illumina MiSeq reads to a nearly identical reference with four reference-guided sequence assemblers before and after read-quality filtering and trimming.** Total numbers of sites and gaps present in consensus sequences calculated from alignments of twelve sets of *Listeria monocytogenes* strain 08-5578 short-read sequence data with four reference-guided assemblers (BWA, MOSAIK, Novoalign, and SMALT) were counted. An *L. monocytogenes* strain 08-5578 chromosome sequence obtained from the National Center for Biotechnology Information archive that is different at three nucleotide positions was used as a reference. The best values (Trim or No trim) for each aligner within each category are bolded.
(PDF)

Table S7 **Total numbers of false positive sites, true positive sites, ambiguous sites, and gaps detected in consensus sequences calculated from alignments of Illumina MiSeq reads to a non-identical reference with four reference-guided sequence assemblers before and after read-quality filtering and trimming.** Total numbers of sites and gaps present in consensus sequences calculated from alignments of twelve sets of *Listeria monocytogenes* strain 08-5578 short-read sequence data with four reference-guided assemblers (BWA, MOSAIK, Novoalign, and SMALT) were counted. An *L. monocytogenes* strain EGD-e chromosome sequence obtained from the National Center for Biotechnology Information archive that is different at 25,347 nucleotide positions was used as a reference. The best values (Trim or No trim) for each aligner within each category are bolded.
(PDF)

Table S8 **Distances of eight** ***Listeria monocytogenes*** **strain 08-5578 consensus sequences calculated from the alignments of four reference-guided assemblers using both** ***L. monocytogenes*** **strains 08-5578 and EGD-e as references.** The best of twelve Illumina MiSeq sequencing runs of *L. monocytogenes* strain 08-5578 genomic DNA was assembled with BWA, MOSAIK, Novoalign, and SMALT using chromosome sequences of both *L. monocytogenes* strains 08-5578 and EGD-e (0.000096% and ∼0.82% distant from the subject at the nucleotide level, respectively), available from the National Center for Biotechnology Information archive, as references. Distances were calculated from 2,735,325 nucleotides with PHYLIP using the Jukes-Cantor method and gamma-distributed weights across sites. Calculated distances of sequences aligned with strain 08-5578 followed by strain EGD-e (*e.g.*, 08-5578/EGD-e) are shown.
(PDF)
