# Supplementary material for: Choice of Reference Sequence and Assembler for Alignment of Listeria monocytogenes Short-Read Sequence Data Greatly Influences Rates of Error in SNP Analyses
Source: PLoS One. 2014 Aug 21;9(8):e104579. doi: 10.1371/journal.pone.0104579 (PMC4140716; doi:10.1371/journal.pone.0104579)
Supplement: Figure S1 — Comparison of consensus sequences calculated from alignments of Illumina MiSeq reads to a nearly identical reference with four reference-guided sequence assemblers both before and after read-quality filtering and trimming. Listeria monocytogenes strain 08-5578 genomic DNA was sequenced twelve times with an Illumina MiSeq benchtop sequencer and the resulting reads were assembled before and after read-quality filtering and trimming with four reference-guided assemblers (BWA, MOSAIK, Novoalign, and SMALT). An L. monocytogenes strain 08-5578 chromosome sequence obtained from the National Center for Biotechnology Information archive that differs at three nucleotide positions was used as a reference. The total numbers of false positive sites (a), true positive sites (b), ambiguous sites (c), and gaps (d) present in all consensus sequences were counted. Error bars were calculated as the square root of the standard deviation of each dataset. (PDF) [file pone.0104579.s001.pdf]

Supporting Information for:

Choice of reference sequence and assembler for alignment of *Listeria monocytogenes* short-read sequence data greatly influences rates of error in SNP analyses

Arthur W. Pightling[1], Nicholas Petronella[2] and Franco Pagotto[1*]

[*] Corresponding author Franco.Pagotto@hc-sc.gc.ca

[1] Listeriosis Reference Service for Canada, Research Division, Bureau of Microbial Hazards, Food Directorate, Health Products and Food Branch, Health Canada, Ottawa, Ontario, Canada

[2] Biostatistics and Modelling Division, Bureau of Food Surveillance and Science Integration, Food Directorate, Health Products and Food Branch, Health Canada, Ottawa, Ontario, Canada

Figure S1: Comparison of consensus sequences calculated from alignments of Illumina MiSeq reads to a nearly identical reference with four reference-guided sequence assemblers both before and after read-quality filtering and trimming. *Listeria monocytogenes* strain 08-5578 genomic DNA was sequenced twelve times with an Illumina MiSeq benchtop sequencer and the resulting reads were assembled before and after read-quality filtering and trimming with four reference-guided assemblers (BWA, MOSAIK, Novoalign, and SMALT). An *L. monocytogenes* strain 08-5578 chromosome sequence obtained from the National Center for Biotechnology Information archive that differs at three nucleotide positions was used as a reference. The total numbers of false positive sites (a), true positive sites (b), ambiguous sites (c), and gaps (d) present in all consensus sequences were counted. Error bars were calculated as the square root of the standard deviation of each dataset.

Figure S1

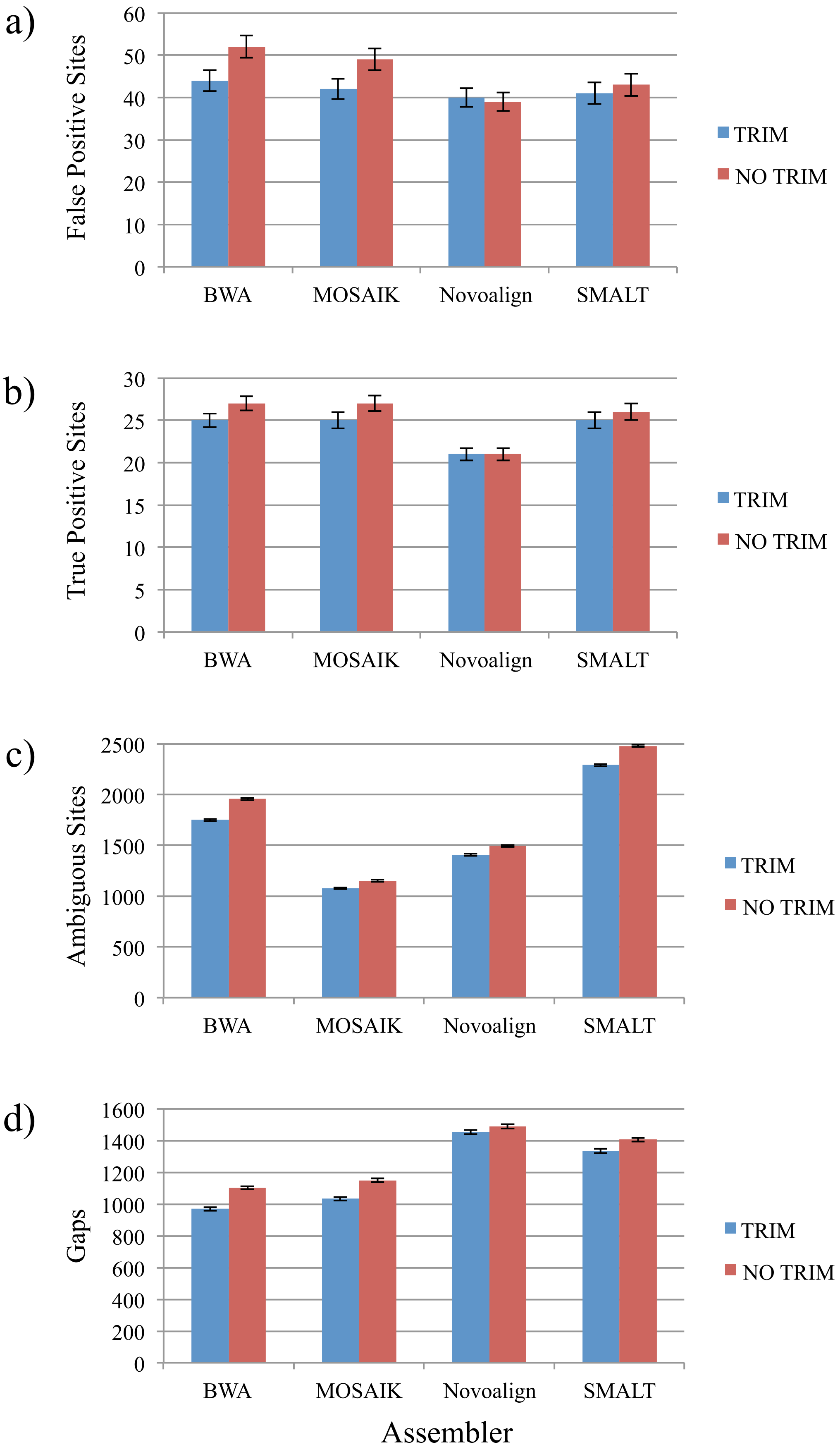
a)
False Positive Sites
60
50
40
30
20
10
0
BWA
MOSAIK
Novoalign
SMALT
TRIM
NO TRIM
b)
True Positive Sites
30
25
20
15
10
5
0
BWA
MOSAIK
Novoalign
SMALT
TRIM
NO TRIM
c)
Ambiguous Sites
2500
2000
1500
1000
500
0
BWA
MOSAIK
Novoalign
SMALT
TRIM
NO TRIM
d)
Gaps
1600
1400
1200
1000
800
600
400
200
0
BWA
MOSAIK
Novoalign
SMALT
TRIM
NO TRIM
Assembler
