# Supplementary material for: Choice of Reference Sequence and Assembler for Alignment of Listeria monocytogenes Short-Read Sequence Data Greatly Influences Rates of Error in SNP Analyses
Source: PLoS One. 2014 Aug 21;9(8):e104579. doi: 10.1371/journal.pone.0104579 (PMC4140716; doi:10.1371/journal.pone.0104579)
Supplement: Figure S2 — Comparison of consensus sequences calculated from alignments of Illumina MiSeq reads to a non-identical reference with four reference-guided sequence assemblers both before and after read-quality filtering and trimming. Listeria monocytogenes strain 08-5578 genomic DNA was sequenced twelve times with an Illumina MiSeq benchtop sequencer and the resulting reads were assembled before and after quality filtering and trimming with four reference-guided assemblers (BWA, MOSAIK, Novoalign, and SMALT). An L. monocytogenes strain EGD-e chromosome sequence obtained from the National Center for Biotechnology Information archive that differs at 25,347 nucleotide positions was used as a reference. The total numbers of false positive sites (a), true positive sites (b), ambiguous sites (c), and gaps (d) present in all consensus sequences were counted. Error bars were calculated as the square root of the standard deviation of each dataset. (PDF) [file pone.0104579.s002.pdf]

Supporting Information for:

Choice of reference sequence and assembler for alignment of *Listeria monocytogenes* short-read sequence data greatly influences rates of error in SNP analyses

Arthur W. Pightling[1], Nicholas Petronella[2] and Franco Pagotto[1*]

[*] Corresponding author Franco.Pagotto@hc-sc.gc.ca

[1] Listeriosis Reference Service for Canada, Research Division, Bureau of Microbial Hazards, Food Directorate, Health Products and Food Branch, Health Canada, Ottawa, Ontario, Canada

[2] Biostatistics and Modelling Division, Bureau of Food Surveillance and Science Integration, Food Directorate, Health Products and Food Branch, Health Canada, Ottawa, Ontario, Canada

Figure S2: Comparison of consensus sequences calculated from alignments of Illumina MiSeq reads to a non-identical reference with four reference-guided sequence assemblers both before and after read-quality filtering and trimming. *Listeria monocytogenes* strain 08-5578 genomic DNA was sequenced twelve times with an Illumina MiSeq benchtop sequencer and the resulting reads were assembled before and after quality filtering and trimming with four reference-guided assemblers (BWA, MOSAIK, Novoalign, and SMALT). An *L. monocytogenes* strain EGD-e chromosome sequence obtained from the National Center for Biotechnology Information archive that differs at 25,347 nucleotide positions was used as a reference. The total numbers of false positive sites (a), true positive sites (b), ambiguous sites (c), and gaps (d) present in all consensus sequences were counted. Error bars were calculated as the square root of the standard deviation of each dataset.

Figure S2

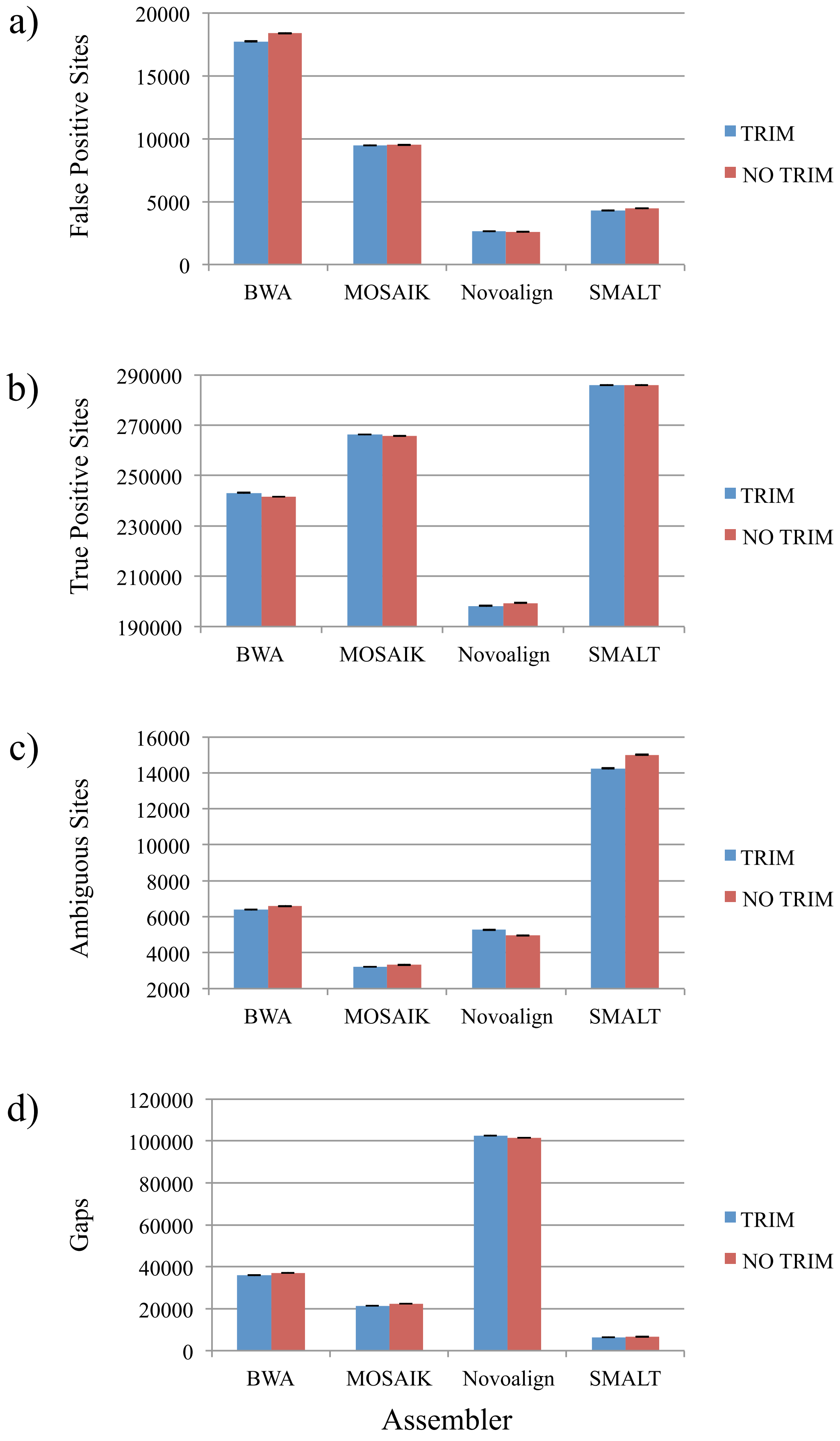
a)
False Positive Sites
20000
15000
10000
5000
0
BWA
MOSAIK
Novoalign
SMALT
TRIM
NO TRIM
b)
True Positive Sites
290000
270000
250000
230000
210000
190000
BWA
MOSAIK
Novoalign
SMALT
TRIM
NO TRIM
c)
Ambiguous Sites
16000
14000
12000
10000
8000
6000
4000
2000
BWA
MOSAIK
Novoalign
SMALT
TRIM
NO TRIM
d)
Gaps
120000
100000
80000
60000
40000
20000
0
BWA
MOSAIK
Novoalign
SMALT
TRIM
NO TRIM
Assembler
