# Supplementary material for: Choice of Reference Sequence and Assembler for Alignment of Listeria monocytogenes Short-Read Sequence Data Greatly Influences Rates of Error in SNP Analyses
Source: PLoS One. 2014 Aug 21;9(8):e104579. doi: 10.1371/journal.pone.0104579 (PMC4140716; doi:10.1371/journal.pone.0104579)
Supplement: Figure S3 — Phylogenetic analysis of eight Listeria monocytogenes strain 08-5578 consensus sequences calculated from the alignments of four reference-guided assemblers using L. monocytogenes strains 08-5578 and EGD-e as references. The best of twelve Illumina MiSeq sequencing runs of L. monocytogenes strain 08-5578 genomic DNA was assembled with BWA, MOSAIK, Novoalign, and SMALT using chromosome sequences of both L. monocytogenes strains 08-5578 and EGD-e (∼0.000096% and ∼0.82% distant from the subject at the nucleotide level, respectively), available from the National Center for Biotechnology Information archive, as references. Trees were calculated from 2,735,325 aligned nucleotides with the Randomized Axelerated Maximum Likelihood tool (RAxML; GTRGAMMA+25+I). The best of 100 bootstrap replicates is shown. (PDF) [file pone.0104579.s003.pdf]

Supporting Information for:

Choice of reference sequence and assembler for alignment of *Listeria monocytogenes* short-read sequence data greatly influences rates of error in SNP analyses

Arthur W. Pightling[1], Nicholas Petronella[2] and Franco Pagotto[1*]

[*] Corresponding author Franco.Pagotto@hc-sc.gc.ca

[1] Listeriosis Reference Service for Canada, Research Division, Bureau of Microbial Hazards, Food Directorate, Health Products and Food Branch, Health Canada, Ottawa, Ontario, Canada

[2] Biostatistics and Modelling Division, Bureau of Food Surveillance and Science Integration, Food Directorate, Health Products and Food Branch, Health Canada, Ottawa, Ontario, Canada

Figure S3: Phylogenetic analysis of eight *Listeria monocytogenes* strain 08-5578 consensus sequences calculated from the alignments of four reference-guided assemblers using *L. monocytogenes* strains 08-5578 and EGD-e as references. The best of twelve Illumina MiSeq sequencing runs of *L. monocytogenes* strain 08-5578 genomic DNA was assembled with BWA, MOSAIK, Novoalign, and SMALT using chromosome sequences of both *L. monocytogenes* strains 08-5578 and EGD-e (~0.000096% and ~0.82% distant from the subject at the nucleotide level, respectively), available from the National Center for Biotechnology Information archive, as references. Trees were calculated from 2,735,325 aligned nucleotides with the Randomized Axelerated Maximum Likelihood tool (RAxML; GTRGAMMA + 25 + I). The best of 100 bootstrap replicates is shown.

Figure S3

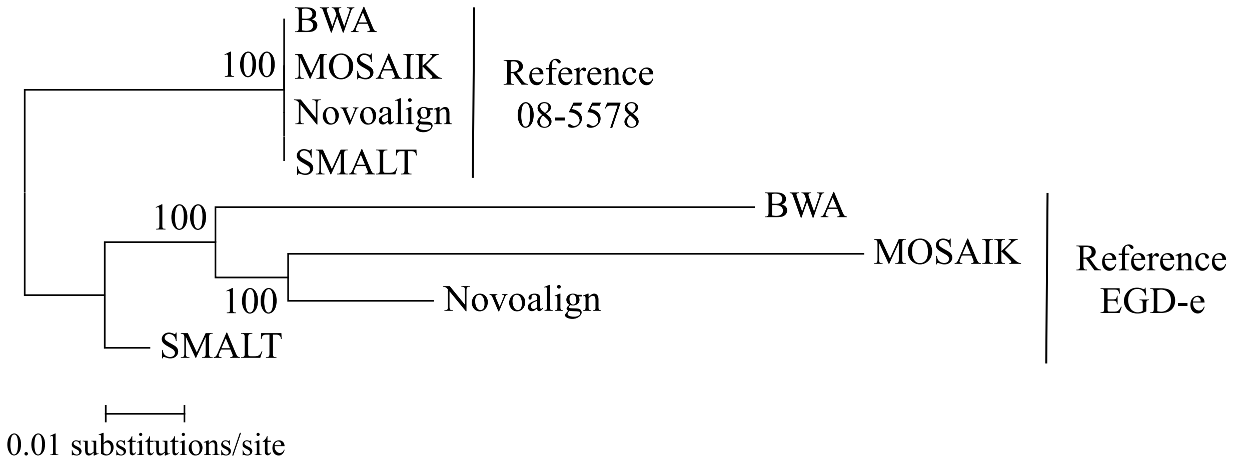
BWA
100
MOSAIK
Novoalign
SMALT
Reference
08-5578
100
BWA
MOSAIK
100
Novoalign
SMALT
Reference
EGD-e
0.01 substitutions/site
