# Supplementary material for: Choice of Reference Sequence and Assembler for Alignment of Listeria monocytogenes Short-Read Sequence Data Greatly Influences Rates of Error in SNP Analyses
Source: PLoS One. 2014 Aug 21;9(8):e104579. doi: 10.1371/journal.pone.0104579 (PMC4140716; doi:10.1371/journal.pone.0104579)
Supplement: Table S1 — Calls used for read-quality trimming and filtering, assembly, and local realignment of Illumina short-read sequences. (PDF) [file pone.0104579.s004.pdf]

Supporting Information for:

Choice of reference sequence and assembler for alignment of *Listeria monocytogenes* short-read sequence data greatly influences rates of error in SNP analyses

Arthur W. Pightling[1], Nicholas Petronella[2] and Franco Pagotto[1*]

[*] Corresponding author Franco.Pagotto@hc-sc.gc.ca

[1] Listeriosis Reference Service for Canada, Research Division, Bureau of Microbial Hazards, Food Directorate, Health Products and Food Branch, Health Canada, Ottawa, Ontario, Canada

[2] Biostatistics and Modelling Division, Bureau of Food Surveillance and Science Integration, Food Directorate, Health Products and Food Branch, Health Canada, Ottawa, Ontario, Canada

Table S1: Calls used for read-quality trimming and filtering, assembly, and local realignment of Illumina short-read sequences.

Function	Application	Calls
Trim and filter reads with PoPoolation	trim-fastq.pl	trim-fastq.pl --fastq-type sanger --input1 file1.fastq --input2 file2.fastq --quality-threshold 20 --min-length 50
Prepare index for BWA alignment	index	bwa index reference.fasta
Prepare reads for BWA alignment	aln	bwa aln reference.fasta file1.fastq > file1.sai; bwa aln reference.fasta file2.fastq > file2.sai
BWA alignment	sampe	bwa sampe reference.fasta file1.sai file2.sai file1.fastq file2.fastq > file.sam
Convert files to MOSAIK format	MosaikBuild	MosaikBuild -fr reference.fasta -oa reference.dat;MosaikBuild -q file1.fastq -q2 file2.fastq -st illumina -mfl 500 -out file.mkb
MOSAIK alignment	MosaikAligner	MosaikAligner -in file.mkb -out file.mka -ia reference.dat -annpe/ MOSAIK/src/networkFile/2.1.78.pe.ann -annse /MOSAIK/src/networkFile/2.1.78.se.ann
Prepare index for Novoalign alignment	novoindex	novoindex reference.nci reference.fasta
Novoalign alignment	novoalign	novoalign --hdrhd off -f file1.fastq file2.fastq -d reference.nci -o SAM > file.sam
Prepare index for SMALT alignment	index	smalt index -k 20 -s 13 reference reference.fasta
SMALT alignment	map	smalt map -f samsoft -o file.sam -n 3 reference file1.fastq file2.fastq
Re-align reads around indels	RealignerTargetCreator/ IndelRealigner	gatk.sh RealignerTargetCreator -I file.bam -R reference.fasta -o file.interfals;gatk.sh IndelRealigner -I file.bam -R reference.fasta --max Consensuses 60 --maxReadsForConsensuses 240 --maxReadsForRealignment 6000 --targetIntervals file.intervals -o file.bam
