# Supplementary material for: Choice of Reference Sequence and Assembler for Alignment of Listeria monocytogenes Short-Read Sequence Data Greatly Influences Rates of Error in SNP Analyses
Source: PLoS One. 2014 Aug 21;9(8):e104579. doi: 10.1371/journal.pone.0104579 (PMC4140716; doi:10.1371/journal.pone.0104579)
Supplement: Table S2 — Numbers of SNPs detected and gaps present in consensus sequences calculated from assemblies of simulated short-read sequence data aligned to references of different genetic distances with four reference-guided assemblers. Ten sets of simulated sequencing reads were generated using a Listeria monocytogenes strain 08-5578 chromosome sequence obtained from the National Center for Biotechnology Information archive as a reference. Nucleotide variants were randomly introduced (101–105) in silico to the 08-5578 chromosome sequence to simulate the presence of SNPs in five reference sequences. The performance of four reference-guided short-read sequence assemblers (BWA, MOSAIK, Novoalign, and SMALT) was assessed by counting the numbers of true SNPs detected and the numbers of gaps present in the consensus sequences generated from alignments of the ten sets of reads. In addition, assembly processing times are provided. The ranges of sites observed are shown with averages in parenthesis. The best values for each category are bolded. (PDF) [file pone.0104579.s005.pdf]

Supporting Information for:

Choice of reference sequence and assembler for alignment of *Listeria monocytogenes* short-read sequence data greatly influences rates of error in SNP analyses

Arthur W. Pightling[1], Nicholas Petronella[2] and Franco Pagotto[1*]

[*] Corresponding author Franco.Pagotto@hc-sc.gc.ca

[1] Listeriosis Reference Service for Canada, Research Division, Bureau of Microbial Hazards, Food Directorate, Health Products and Food Branch, Health Canada, Ottawa, Ontario, Canada

[2] Biostatistics and Modelling Division, Bureau of Food Surveillance and Science Integration, Food Directorate, Health Products and Food Branch, Health Canada, Ottawa, Ontario, Canada

Table S2: Numbers of SNPs detected and gaps present in consensus sequences calculated from assemblies of simulated short-read sequence data aligned to references of different genetic distances with four reference-guided assemblers. Ten sets of simulated sequencing reads were generated using a *Listeria monocytogenes* strain 08-5578 chromosome sequence obtained from the National Center for Biotechnology Information archive as a reference. Nucleotide variants were randomly introduced (10^1-10^5) *in silico* to the 08-5578 chromosome sequence to simulate the presence of SNPs in five reference sequences. The performance of four reference-guided short-read sequence assemblers (BWA, MOSAIK, Novoalign, and SMALT) was assessed by counting the numbers of true SNPs detected and the numbers of gaps present in the consensus sequences generated from alignments of the ten sets of reads. In addition, assembly processing times are provided. The ranges of sites observed are shown with averages in parenthesis. The best values for each category are bolded.

	10 (0.00032%)			100 (0.0032%)		
	SNPs Detected	Gaps	Time (seconds)	SNPs Detected	Gaps	Time (seconds)
BWA	**10 (10.00)**	**0 (0.00)**	40.42-40.73 (40.60)	**99 (99.00)**	**1 (1.00)**	40.27-40.68 (40.47)
MOSAIK	**10 (10.00)**	22-24 (23.00)	340.87-343.16 (342.36)	98 (98.00)	25-26 (25.30)	341.23-342.78 (342.13)
Novoalign	**10 (10.00)**	29-31 (30.00)	1014.22-1045.13 (1035.27)	**99 (99.00)**	30-34 (32.10)	1040.40-1044.52 (1042.28)
SMALT	**10 (10.00)**	0-1 (0.90)	**33.30-34.02 (33.61)**	**99 (99.00)**	**1 (1.00)**	**33.42-34.00 (33.60)**

Table S2 (continued): Numbers of SNPs detected and gaps present in consensus sequences calculated from assemblies of simulated short-read sequence data aligned to references of different genetic distances with four reference-guided assemblers. Ten sets of simulated sequencing reads were generated using a *Listeria monocytogenes* strain 08-5578 chromosome sequence obtained from the National Center for Biotechnology Information archive as a reference. Nucleotide variants were randomly introduced (10^1-10^5) *in silico* to the 08-5578 chromosome sequence to simulate the presence of SNPs in five reference sequences. The performance of four reference-guided short-read sequence assemblers (BWA, MOSAIK, Novoalign, and SMALT) was assessed by counting the numbers of true SNPs detected and the numbers of gaps present in the consensus sequences generated from alignments of the ten sets of reads. In addition, assembly processing times are provided. The ranges of sites observed are shown with averages in parenthesis. The best values for each category are bolded.

	1000 (0.032%)			10000 (0.32%)		
	SNPs Detected	Gaps	Time (seconds)	SNPs Detected	Gaps	Time (seconds)
BWA	988-989 (988.20)	1 (1.00)	40.44-40.89 (40.66)	9911-9921 (9914.60)	**1 (1.00)**	43.49-44.72 (43.833)
MOSAIK	988-989 (988.20)	32-34 (33.00)	341.66-343.15 (342.30)	9912-9922 (9916.00)	66-74 (69.10)	342.43-344.21 (343.18)
Novoalign	**988-990 (988.60)**	43-47 (45.00)	1019.44-1051.49 (1041.24)	**9929-9940 (9932.80)**	111-118 (114.40)	1103.80-1132.42 (1127.53)
SMALT	988-989 (988.10)	**0-1 (0.90)**	**33.52-33.77 (33.65)**	9907-9919 (9911.80)	**1 (1.00)**	**33.24-33.69 (33.56)**

Table S2 (continued): Numbers of SNPs detected and gaps present in consensus sequences calculated from assemblies of simulated short-read sequence data aligned to references of different genetic distances with four reference-guided assemblers. Ten sets of simulated sequencing reads were generated using a *Listeria monocytogenes* strain 08-5578 chromosome sequence obtained from the National Center for Biotechnology Information archive as a reference. Nucleotide variants were randomly introduced (10^1-10^5) *in silico* to the 08-5578 chromosome sequence to simulate the presence of SNPs in five reference sequences. The performance of four reference-guided short-read sequence assemblers (BWA, MOSAIK, Novoalign, and SMALT) was assessed by counting the numbers of true SNPs detected and the numbers of gaps present in the consensus sequences generated from alignments of the ten sets of reads. In addition, assembly processing times are provided. The ranges of sites observed are shown with averages in parenthesis. The best values for each category are bolded.

	100000 (3.2%)		
	SNPs Detected	Gaps	Time (seconds)
BWA	94499-99585 (94538.10)	19-53 (39.20)	156.77-198.62 (186.56)
MOSAIK	94965-95043 (95007.20)	604-632 (624.00)	395.69-446.55 (434.95)
Novoalign	93861-93953 (93919.20)	1957-2125 (2024.60)	2439.17-2492.68 (2470.05)
SMALT	**94997-95051 (95021.30)**	**1-5 (1.90)**	**46.56-81.72 (68.59)**
