# Supplementary material for: Choice of Reference Sequence and Assembler for Alignment of Listeria monocytogenes Short-Read Sequence Data Greatly Influences Rates of Error in SNP Analyses
Source: PLoS One. 2014 Aug 21;9(8):e104579. doi: 10.1371/journal.pone.0104579 (PMC4140716; doi:10.1371/journal.pone.0104579)
Supplement: Table S3 — Summary statistics describing three sequencing runs of four Listeria monocytogenes strain 08-5578 genomic DNA samples on an Illumina MiSeq benchtop sequencer. Genomic DNA was extracted from an L. monocytogenes strain 08-5578 culture grown from a single colony. The sample was then divided into four subsamples that were indexed and sequenced three times. (PDF) [file pone.0104579.s006.pdf]

# Supporting Information for:

## Choice of reference sequence and assembler for alignment of *Listeria monocytogenes* short-read sequence data greatly influences rates of error in SNP analyses

Arthur W. Pightling[1], Nicholas Petronella[2] and Franco Pagotto[1*]

[*] Corresponding author Franco.Pagotto@hc-sc.gc.ca

[1] Listeriosis Reference Service for Canada, Research Division, Bureau of Microbial Hazards, Food Directorate, Health Products and Food Branch, Health Canada, Ottawa, Ontario, Canada

[2] Biostatistics and Modelling Division, Bureau of Food Surveillance and Science Integration, Food Directorate, Health Products and Food Branch, Health Canada, Ottawa, Ontario, Canada

**Table S3: Summary statistics describing three sequencing runs of four *Listeria monocytogenes* strain 08-5578 genomic DNA samples on an Illumina MiSeq benchtop sequencer.** Genomic DNA was extracted from an *L. monocytogenes* strain 08-5578 culture grown from a single colony. The sample was then divided into four subsamples that were indexed and sequenced three times.

| Run | Yield | Average Cluster Density (k/mm2) | Total Reads | Reads Passing Filter |
|---|---|---|---|---|
| 1 | 3.96GB | 478.5 | 9,357,411 | 8,506,127 |
| 2 | 1.16GB | 178.5 | 5,077,244 | 2,302,912 |
| 3 | 7.76GB | 1075.0 | 19,068,628 | 16,320,666 |
