# Supplementary material for: Choice of Reference Sequence and Assembler for Alignment of Listeria monocytogenes Short-Read Sequence Data Greatly Influences Rates of Error in SNP Analyses
Source: PLoS One. 2014 Aug 21;9(8):e104579. doi: 10.1371/journal.pone.0104579 (PMC4140716; doi:10.1371/journal.pone.0104579)
Supplement: Table S4 — Numbers of false positive sites, true positive sites, ambiguous sites, and gaps detected in consensus sequences calculated from alignments of Illumina short-read data to a nearly identical reference with four reference-guided assemblers. The ability of four reference-guided short-read sequence assemblers (BWA, MOSAIK, Novoalign, and SMALT) to align Listeria monocytogenes strain 08-5578 genome sequence data was assessed by aligning twelve sets of reads to a reference chromosome sequence that differs by three nucleotides. The ranges of events observed are shown with averages in parentheses. The values for all twelve datasets are provided as well as those with 50-fold or greater coverage. The best values for each category are bolded. (PDF) [file pone.0104579.s007.pdf]

Supporting Information for:

Choice of reference sequence and assembler for alignment of *Listeria monocytogenes* short-read sequence data greatly influences rates of error in SNP analyses

Arthur W. Pightling[1], Nicholas Petronella[2] and Franco Pagotto[1*]

[*] Corresponding author Franco.Pagotto@hc-sc.gc.ca

[1] Listeriosis Reference Service for Canada, Research Division, Bureau of Microbial Hazards, Food Directorate, Health Products and Food Branch, Health Canada, Ottawa, Ontario, Canada

[2] Biostatistics and Modelling Division, Bureau of Food Surveillance and Science Integration, Food Directorate, Health Products and Food Branch, Health Canada, Ottawa, Ontario, Canada

Table S4: Numbers of false positive sites, true positive sites, ambiguous sites, and gaps detected in consensus sequences calculated from alignments of Illumina short-read data to a nearly identical reference with four reference-guided assemblers. The ability of four reference-guided short-read sequence assemblers (BWA, MOSAIK, Novoalign, and SMALT) to align *Listeria monocytogenes* strain 08-5578 genome sequence data was assessed by aligning twelve sets of reads to a reference chromosome sequence that differs by three nucleotides. The ranges of events observed are shown with averages in parentheses. The values for all twelve datasets are provided as well as those with 50-fold or greater coverage. The best values for each category are bolded.

	False Positive Sites		True Positive Sites		Ambiguous Sites		Gaps	
	Total	≥ 50X	Total	≥ 50X	Total	≥ 50X	Total	≥ 50X
BWA	0-22 (3.75)	1 (1.00)	**1-3 (2.33)**	**3 (3.00)**	13-363 (147.00)	13-14 (13.5)	**4-322 (81.08)**	4 (4.00)
MOSAIK	0-19 (3.50)	**0 (0.00)**	**1-3 (2.33)**	**3 (3.00)**	**12-277 (90.75)**	**12-14 (13.00)**	8-341 (86.75)	4 (4.00)
Novoalign	**0-15 (3.33)**	**0 (0.00)**	1-3 (1.92)	2 (2.00)	10-312 (119.00)	11-21 (14.67)	13-469 (122.25)	13-19 (16.67)
SMALT	0-20 (3.42)	**0 (0.00)**	**1-3 (2.33)**	**3 (3.00)**	17-431 (192.25)	17-23 (19.33)	2-424 (111.42)	**2-6 (3.67)**
