# Supplementary material for: Choice of Reference Sequence and Assembler for Alignment of Listeria monocytogenes Short-Read Sequence Data Greatly Influences Rates of Error in SNP Analyses
Source: PLoS One. 2014 Aug 21;9(8):e104579. doi: 10.1371/journal.pone.0104579 (PMC4140716; doi:10.1371/journal.pone.0104579)
Supplement: Table S5 — Numbers of false positive sites, true positive sites, ambiguous sites, and gaps detected in consensus sequences calculated from alignments of Illumina short-read data to a non-identical reference with four reference-guided assemblers. The ability of four reference-guided short-read sequence assemblers (BWA, MOSAIK, Novoalign, and SMALT) to align Listeria monocytogenes strain 08-5578 genome sequence data was assessed by aligning twelve sets of reads to a reference chromosome sequence (strain EGD-e) that differs by 25,347 nucleotides. The ranges of events observed are shown with averages in parentheses. The values for all twelve datasets are provided as well as those with 50-fold or greater coverage. The best values for each category are bolded. (PDF) [file pone.0104579.s008.pdf]

Supporting Information for:

Choice of reference sequence and assembler for alignment of *Listeria monocytogenes* short-read sequence data greatly influences rates of error in SNP analyses

Arthur W. Pightling[1], Nicholas Petronella[2] and Franco Pagotto[1*]

[*] Corresponding author Franco.Pagotto@hc-sc.gc.ca

[1] Listeriosis Reference Service for Canada, Research Division, Bureau of Microbial Hazards, Food Directorate, Health Products and Food Branch, Health Canada, Ottawa, Ontario, Canada

[2] Biostatistics and Modelling Division, Bureau of Food Surveillance and Science Integration, Food Directorate, Health Products and Food Branch, Health Canada, Ottawa, Ontario, Canada

Table S5: Numbers of false positive sites, true positive sites, ambiguous sites, and gaps detected in consensus sequences calculated from alignments of Illumina short-read data to a non-identical reference with four reference-guided assemblers. The ability of four reference-guided short-read sequence assemblers (BWA, MOSAIK, Novoalign, and SMALT) to align *Listeria monocytogenes* strain 08-5578 genome sequence data was assessed by aligning twelve sets of reads to a reference chromosome sequence (strain EGD-e) that differs by 25,347 nucleotides. The ranges of events observed are shown with averages in parentheses. The values for all twelve datasets are provided as well as those with 50-fold or greater coverage. The best values for each category are bolded.

	False Positive Sites		True Positive Sites		Ambiguous Sites		Gaps	
	Total	≥ 50X	Total	≥ 50X	Total	≥ 50X	Total	≥ 50X
BWA	791-2701 (1477.17)	791-952 (871.50)	16884-21792 (20262.08)	21753-21792 (21772.50)	251-932 (533.50)	251-284 (267.50)	1938-5305 (3004.10)	1938-1978 (1958.00)
MOSAIK	657-1053 (787.92)	657-733 (695.00)	21427-22696 (22185.67)	22614-22696 (22655.00)	**141-527 (268.00)**	165-188 (176.50)	1120-2629 (1787.80)	1120-1249 (1184.50)
Novoalign	**194-249 (218.83)**	**220 (220.00)**	14734-19722 (16518.00)	19722 (19722.00)	88-810 (439.00)	**129 (129.00)**	5058-10718 (8549.20)	5058 (5058.00)
SMALT	300-414 (356.67)	302-330 (314.33)	**23511-24012 (23820.92)**	**23934-24012 (23978.33)**	801-1608 (1186.75)	801-832 (817.33)	**218-1175 (534.25)**	**218-287 (245.33)**
