# Supplementary material for: Choice of Reference Sequence and Assembler for Alignment of Listeria monocytogenes Short-Read Sequence Data Greatly Influences Rates of Error in SNP Analyses
Source: PLoS One. 2014 Aug 21;9(8):e104579. doi: 10.1371/journal.pone.0104579 (PMC4140716; doi:10.1371/journal.pone.0104579)
Supplement: Table S6 — Total numbers of false positive sites, true positive sites, ambiguous sites, and gaps detected in consensus sequences calculated from alignments of Illumina MiSeq reads to a nearly identical reference with four reference-guided sequence assemblers before and after read-quality filtering and trimming. Total numbers of sites and gaps present in consensus sequences calculated from alignments of twelve sets of Listeria monocytogenes strain 08-5578 short-read sequence data with four reference-guided assemblers (BWA, MOSAIK, Novoalign, and SMALT) were counted. An L. monocytogenes strain 08-5578 chromosome sequence obtained from the National Center for Biotechnology Information archive that is different at three nucleotide positions was used as a reference. The best values (Trim or No trim) for each aligner within each category are bolded. (PDF) [file pone.0104579.s009.pdf]

Supporting Information for:

Choice of reference sequence and assembler for alignment of *Listeria monocytogenes* short-read sequence data greatly influences rates of error in SNP analyses

Arthur W. Pightling[1], Nicholas Petronella[2] and Franco Pagotto[1*]

[*] Corresponding author Franco.Pagotto@hc-sc.gc.ca

[1] Listeriosis Reference Service for Canada, Research Division, Bureau of Microbial Hazards, Food Directorate, Health Products and Food Branch, Health Canada, Ottawa, Ontario, Canada

[2] Biostatistics and Modelling Division, Bureau of Food Surveillance and Science Integration, Food Directorate, Health Products and Food Branch, Health Canada, Ottawa, Ontario, Canada

Table S6: Total numbers of false positive sites, true positive sites, ambiguous sites, and gaps detected in consensus sequences calculated from alignments of Illumina MiSeq reads to a nearly identical reference with four reference-guided sequence assemblers before and after read-quality filtering and trimming. Total numbers of sites and gaps present in consensus sequences calculated from alignments of twelve sets of *Listeria monocytogenes* strain 08-5578 short-read sequence data with four reference-guided assemblers (BWA, MOSAIK, Novoalign, and SMALT) were counted. An *L. monocytogenes* strain 08-5578 chromosome sequence obtained from the National Center for Biotechnology Information archive that is different at three nucleotide positions was used as a reference. The best values (Trim or No trim) for each aligner within each category are bolded.

	False Positive Sites		True Positive Sites		Ambiguous Sites		Gaps	
	Trim	No trim	Trim	No trim	Trim	No trim	Trim	No trim
BWA	**44**	52	25	**27**	**1589**	1779	**951**	1080
MOSAIK	**42**	49	25	**27**	**987**	1051	**1014**	1123
Novoalign	40	**39**	**21**	**21**	**1295**	1373	**1413**	1456
SMALT	**41**	43	25	**26**	**2067**	2231	**1305**	1372
