# Supplementary material for: Choice of Reference Sequence and Assembler for Alignment of Listeria monocytogenes Short-Read Sequence Data Greatly Influences Rates of Error in SNP Analyses
Source: PLoS One. 2014 Aug 21;9(8):e104579. doi: 10.1371/journal.pone.0104579 (PMC4140716; doi:10.1371/journal.pone.0104579)
Supplement: Table S8 — Distances of eight Listeria monocytogenes strain 08-5578 consensus sequences calculated from the alignments of four reference-guided assemblers using both L. monocytogenes strains 08-5578 and EGD-e as references. The best of twelve Illumina MiSeq sequencing runs of L. monocytogenes strain 08-5578 genomic DNA was assembled with BWA, MOSAIK, Novoalign, and SMALT using chromosome sequences of both L. monocytogenes strains 08-5578 and EGD-e (0.000096% and ∼0.82% distant from the subject at the nucleotide level, respectively), available from the National Center for Biotechnology Information archive, as references. Distances were calculated from 2,735,325 nucleotides with PHYLIP using the Jukes-Cantor method and gamma-distributed weights across sites. Calculated distances of sequences aligned with strain 08-5578 followed by strain EGD-e (e.g., 08-5578/EGD-e) are shown. (PDF) [file pone.0104579.s011.pdf]

Supporting Information for:

Choice of reference sequence and assembler for alignment of *Listeria monocytogenes* short-read sequence data greatly influences rates of error in SNP analyses

Arthur W. Pightling[1], Nicholas Petronella[2] and Franco Pagotto[1*]

[*] Corresponding author Franco.Pagotto@hc-sc.gc.ca

[1] Listeriosis Reference Service for Canada, Research Division, Bureau of Microbial Hazards, Food Directorate, Health Products and Food Branch, Health Canada, Ottawa, Ontario, Canada

[2] Biostatistics and Modelling Division, Bureau of Food Surveillance and Science Integration, Food Directorate, Health Products and Food Branch, Health Canada, Ottawa, Ontario, Canada

Table S8: Distances of eight *Listeria monocytogenes* strain 08-5578 consensus sequences calculated from the alignments of four reference-guided assemblers using both *L. monocytogenes* strains 08-5578 and EGD-e as references. The best of twelve Illumina MiSeq sequencing runs of *L. monocytogenes* strain 08-5578 genomic DNA was assembled with BWA, MOSAIK, Novoalign, and SMALT using chromosome sequences of both *L. monocytogenes* strains 08-5578 and EGD-e (0.000096% and ~0.82% distant from the subject at the nucleotide level, respectively), available from the National Center for Biotechnology Information archive, as references. Distances were calculated from 2,735,325 nucleotides with PHYLIP using the Jukes-Cantor method and gamma-distributed weights across sites. Calculated distances of sequences aligned with strain 08-5578 followed by strain EGD-e (*e.g.,* 08-5578 / EGD-e) are shown.

	BWA	MOSAIK	Novoalign
MOSAIK	0.000000 / 0.000353		
Novoalign	0.000000 / 0.000170	0.000000 / 0.000148	
SMALT	0.000000 / 0.000222	0.000000 / 0.000251	0.000000 / 0.000078
